# Root surface roughness evaluation following application of different periodontal instruments and Er:YAG laser: A profilometry and SEM study

**DOI:** 10.1007/s10103-024-04025-y

**Published:** 2024-04-07

**Authors:** Gizem İnce Kuka, Hare Gürsoy

**Affiliations:** Department of Periodontology, Hamidiye Dental Faculty, University of Health Sciences, Istanbul, Turkey

**Keywords:** Er-YAG laser, Ultrasonic tip, Stainless steel curette, Surface roughness

## Abstract

**Aim:**

The aim of the present study was to evaluate the efficacy of 30^°^-angled Er:YAG laser tip and different periodontal instruments on root surface roughness and morphology in vitro.

**Methods:**

Eighteen bovine teeth root without carious lesion were decoronated from the cementoenamel junction and seperated longitidunally. A total of 36 obtained blocks were mounted in resin blocks and polished with silicon carbide papers under water irrigation. These blocks were randomly assigned into 3 treatment groups. In Group 1, 30°-angled Er:YAG laser (2.94 μm) tip was applied onto the blocks with a 20 Hz, 120 mJ energy output under water irrigation for 20 s. In Groups 2 and 3, the same treatment was applied to the blocks with new generation ultrasonic tip and conventional curette apico-coronally for 20 s with a sweeping motion. Surface roughness and morphology were evaluated before and after instrumentation with a profilometer and SEM, respectively.

**Results:**

After instrumentation, profilometric analysis revealed significantly higher roughness values compared to baseline in all treatment groups(*p* < 0.05). Laser group revealed the roughest surface morphology followed by conventional curette and new generation ultrasonic tip treatment groups (*p* < 0.05). In SEM analysis, irregular surfaces and crater defects were seen more frequently in the laser group.

**Conclusion:**

Results of the study showed that the use of new generation ultrasonic tip was associated with smoother surface morphology compared to 30°-angled Er-YAG laser tip and conventional curette. Further in vitro and in vivo studies with an increased sample size are necessary to support the present study findings.

## Introduction

Periodontitis is the most frequently encountered chronic inflammatory disease of the oral cavity with a world-wide distribution [1, 2]. Non-surgical periodontal treatment aims to achieve biologically-acceptable root surface by removal of the dental biofilm and hard deposits which act as an etiological factor for the development of periodontal disease [3]. During treatment procedures, it is crucial to eliminate these local etiological factors without damaging root surface morphology. Following root surface debridement, possible induction of surface roughness may facilitate dental biofilm accumulation, and thereby initiate inflammation characterized by the increase of the gingival crevicular fluid volume and erythema formation [4, 5]. Over the counter, many hand instruments and ultrasonic devices are available for the use of root surface debridement with contradictory results in the literature regarding their treatment efficacy and ease of use [6, 7]. In the past, hand instruments were stated to be a gold standard in non-surgical treatment of periodontal diseases. However, with the development of technology, usage of ultrasonic tips has gained popularity in terms of improved patient comfort and less fatique formation to the clinician during treatment procedures with efficient root surface debridement properties [5, 6, 8]. The use of different types of periodontal instruments in non-surgical periodontal treatment has different disadvantages such as the loss of the instrument sharpness within time, limited tactile sensitivity and requirement of an adequate experience in order to achieve ideal treatment outcomes [9]. In recent years, Er:YAG lasers are also stated to remove calculus and smear layer from the diseased root surfaces without harming the neighbouring soft tissues [6, 10, 11]. The aim of the present study was to compare root surface roughness and morphology following the use of Er:YAG laser 30°-angled tip, new generation ultrasonic tip and conventional curette in vitro. The null hypothesis of the study is that neither root surface roughness nor morphology will differ among the treatment groups following instrumentation.

## Materials & methods

Sample size calculation was performed according to Amid R et al. [12] Effect size was taken as 0.65 and alpha error as 0.05 to achieve 80% power, and 12 samples were needed for each treatment group.

Eighteen non-carious single-rooted bovine teeth, which were separated from their crowns and halved longitudinally, were mounted in resin acrylic blocks with 5-mm of thickness. Since the study was conducted on the extracted bovine teeth, neither ethical approval nor informed consent for the extraction was required. A total of 36 samples were polished under water irrigation with silicon carpide papers randomly assigned into 3 different treatment groups as described below.

### Group 1 (*n* = 12)

Er:YAG laser with 2.94 μm wavelength (VersaWave, Delight; Hoya-Con Bio, Fremont, Canada). 30°-angled tip was used along the root surface for 20 s under water irrigation in contact mode at 120 mJ and 20 Hz parameters.

### Group 2 (*n* = 12)

New generation ultrasonic tip (Newtron^®^ P5XS BLED H3, Acteon, France) was applied along the root surface, providing ≤ 15° angle between the scaler tip and root surface by adaptation of the terminal 2–3 mm of the tip of the instrument under irrigation at medium power setting for 20 s [2]. 

### Group 3 (*n* = 12)

Conventional stainless steel curette (Bliss Gracey 5/6, Acteon, France) was used along the root surface for 20 s.

Root surface roughness evaluations were performed with a profilometer (Perthometer M1 Mahr, Göttingen, Germany). A total of 5 measurements were calculated in each sample surface at different directions and locations with 0.25 mm cut off and 0.8 mm trace-lengths, having 0.1 mm stylus speed per second. Average root surface roughness (Ra) (mean between peaks and valleys of the surface profile) [9] was evaluated before and after instrumentations, and recorded as an average of these 5 measurements as described by Gursoy et al. [10] Mean roughness height (Rz) (mean of five peaks and five valley height in each cutoff) [9] and maximum roughness depth (Rmax) (the maximum peak to valley of a profile) [13] were evaluated following instrumentations.

In each treatment group, representative scanning electron microscobe (SEM) images were obtained following instrumentations. Samples were fixed in 2.5% glutaraldehyde and 0.1 M phosphate-buffered saline for 24 h at room temperature, then washed with distilled water and air-dried. Later on, samples were coated with 20 nm gold layer in a sputter coater and placed in the vacuum chamber of SEM (JSM 6335 F; JEOL-USA, Inc., Peabody, MA, USA) at a 5 kV accelerating voltage. SEM microphotographs were taken from each sample at 2000 magnification [5]. 

### Statistical analysis

Statistical Package for the Social Sciences (SPSS 24) software programme was used for statistical analysis. Quantitative data were recorded as mean, standard deviation, median, minimum and maximum. Intragroup differences were evaluated with Wilcoxon signed rank test. Comparison of 3 groups were evaluated with Kruskall Wallis test. Further double intergroup comparisons were performed with Mann Whitney U test. Significance level was taken as 0.05.

## Results

Baseline mean surface roughness (Ra) of the prepared root samples revealed similar values, and no significant differences were detected (*p* = 0.655). Following instrumentations, Ra values increased significantly in each treatment group (*p* = 0.001) (Table 1).

**Table 1:** Comparisons of Ra values at baseline and following instrumentations

	30°-angled Er:YAG laser tip	New generation ultrasonic tip	Stainless steel curette	*P* ^b^
	Mean ± SD (Median)	Mean ± SD (Median)	Mean ± SD (Median)	
Baseline	0.05 ± 0.02 (0.05)	0.04 ± 0.01 (0.04)	0.05 ± 0.06 (0.05)	0.655
Instrumentation	0.41 ± 0.06 (0.42)	0.14 ± 0.02 (0.14)	0.18 ± 0.05 (0.16)	**0.040**
*p* ^a^	**0.001**	**0.001**	**0.001**	

^a^ Wilcoxon Signed Rank Test, ^b^Kruskall Wallis Test, *p* < 0.05

Among the treatment groups, 30°-angled Er:YAG laser tip revealed the roughest surface compared to new-generation ultrasonic tip and stainless steel curette groups (*p* < 0.05) (Table 2). New-generation ultrasonic tip exhibited the smoothest surface followed by stainless steel curette. However, further double comparisons of Rz and Rmax values revealed no significant differences between these groups following instrumentations (*p* = 0.550,*p* = 0.120) (Table 3).

**Table 2:** Inter-group comparison of the parameters (Rz and Rmax) following instrumentations

	30°-angled Er:YAG laser tip	New generation ultrasonic tip	Stainless steel curette	*p*
	Mean ± SD (Median)	Mean ± SD (Median)	Mean ± SD (Median)	
Rz	2.18 ± 0.34 (2.09)	1.08 ± 0.39 (0.93)	1.19 ± 0.37 (1.27)	**0.001**
Rmax	2.97 ± 0.6 (2.95)	1.64 ± 0.66 (1.45)	2.39 ± 0.41 (2.38)	**0.001**

Kruskall Wallis Test, *p* < 0.05

**Table 3:** Inter-group double comparison of the parameters (Ra, Rz, Rmax) following instrumentations

	30°-angled Er:YAG Laser tip - New generation ultrasonic tip	30°-angled Er:YAG Laser tip - Stainless steel curette	New generation ultrasonic tip - Stainless steel curette
Ra	**0.001**	**0.001**	**0.019**
Rz	**0.001**	**0.001**	0.550
Rmax	**0.001**	**0.001**	0.120

Mann Whitney U test, *p* < 0.05

**Fig. 1:**
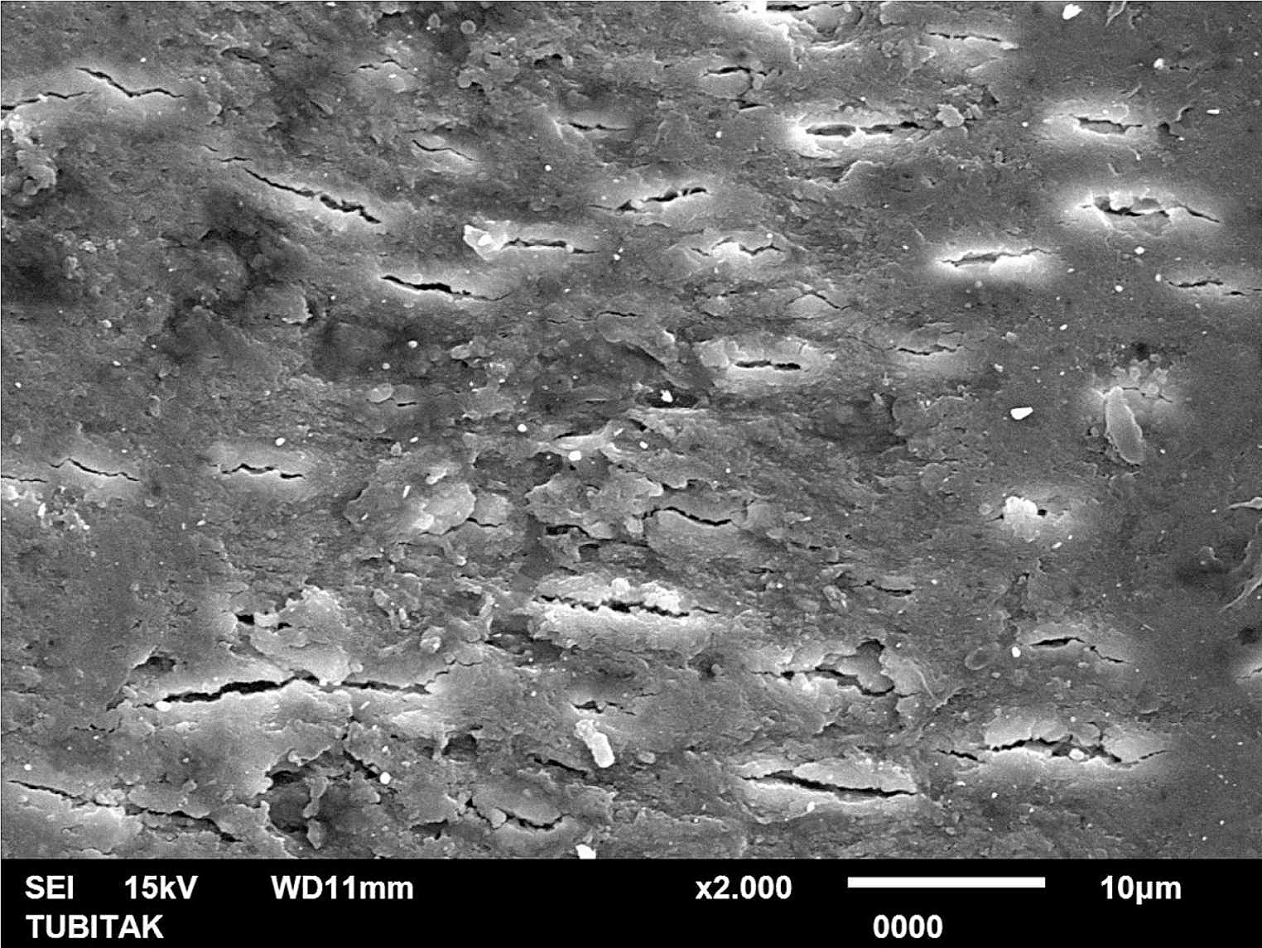
SEM image of the 30°-angled Er:YAG laser tip-applied treatment group (x2000 magnification)

**Fig. 2:**
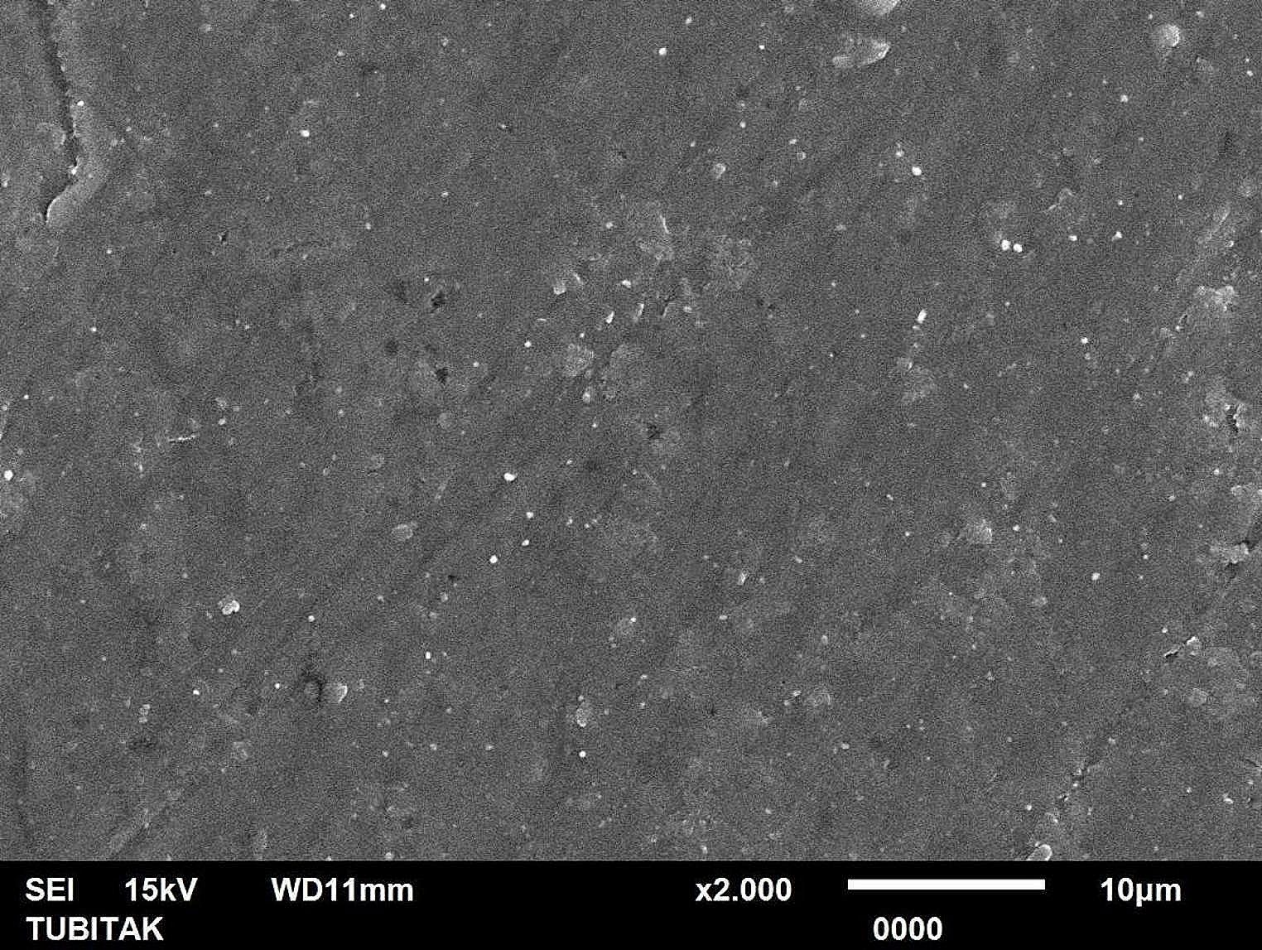
SEM image of the new generation ultrasonic tip-applied treatment group (x2000 magnification)

**Fig. 3:**
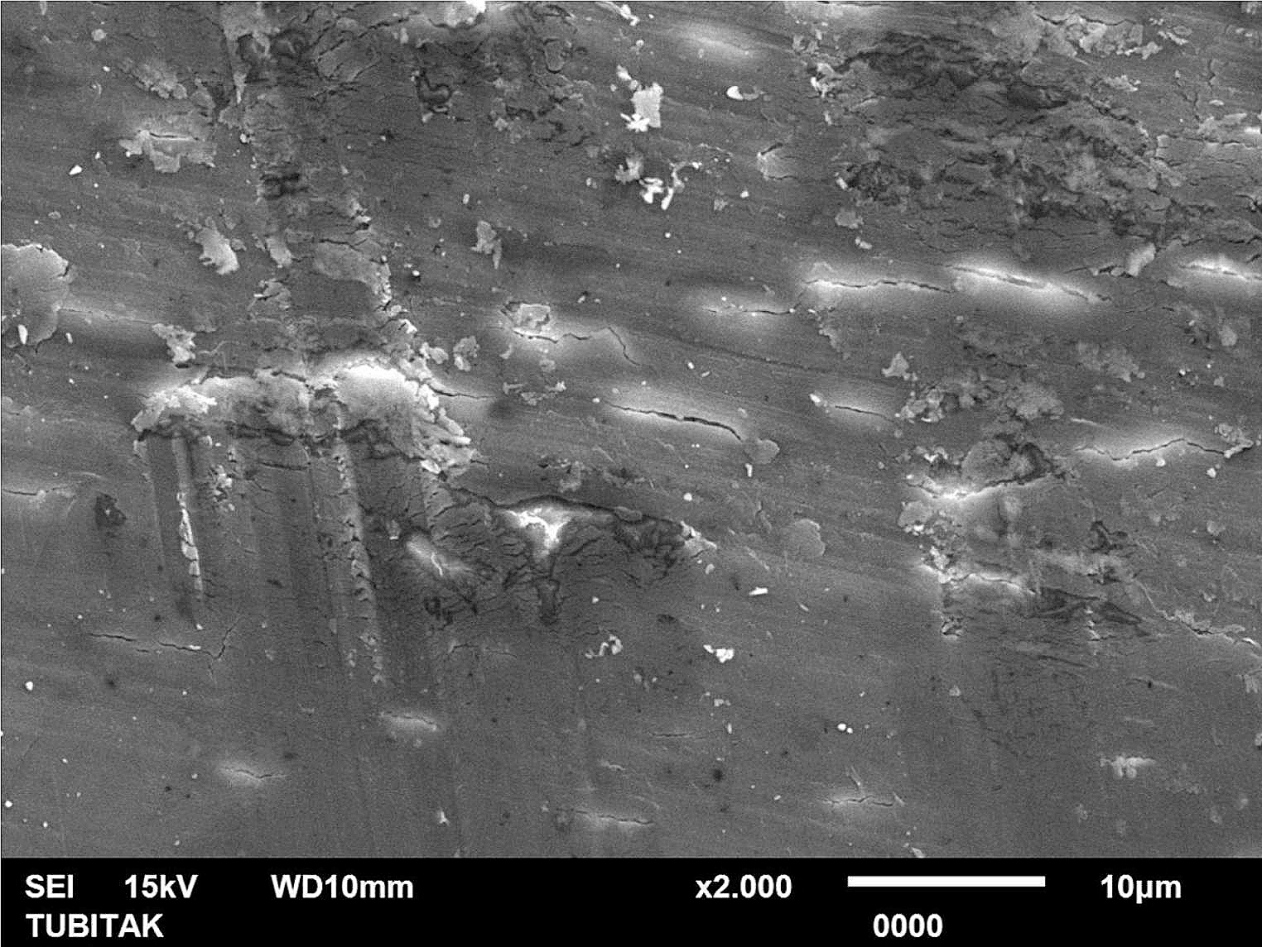
SEM image of the conventional curette-applied treatment group (x2000 magnification)

In SEM microphotographs, similar to profilometer results, 30°-angled Er:YAG laser tip revealed the most irregular surface together with crater formations (Fig. 1). In stainless steel treatment group, surface scratches were more frequent and deep compared to new generation ultrasonic tip group, which exhibited the homogenous surface morphology (Figs. 2 and 3).

## Discussion

In non-surgical treatment of periodontitis, the most important factor for long-term success of the treatment is to obtain a clean and biologically acceptable root surface [3]. For years, stainless steel instruments, which are referred as gold standard, have been frequently used for root surface debridement in clinical periodontology practice [10]. Recently, new generation ultrasonic tips, which enhance the clinician ergonomy by it’s ease of use, have been introduced to the counter [9]. A limited number of studies comparing new ultrasonic tips with stainless steel curettes report controversial results in treatment efficacy, but state that ultrasonics are advantageous in terms of ease of use and user comfort [9, 12, 14–19]. Nowadays, use of ultrasonics and angled laser tips in root surface debridement has gained popularity since conventional curettes have disadvantages including the loss of sharpness within time, need of sharpening and difficulties in manipulation [6, 10, 16, 17, 20, 21]. Findings of the present study revealed that the new generation ultrasonic tip provided smoother root surface morphology compared to the conventional curette. Kumar et al. [15] have evaluated the root surface roughness formation following the use of curettes and ultrasonic tips with different power settings. Similar to our findings, the smoothest surface has been obtained by the use of ultrasonics at medium power setting, whereas high power setting and curette have induced similar and rougher surface morphology compared to the use of ultrasonics at medium power setting.

In the presence of deep periodontal pockets, angled laser tips are reported to better reach to the base of the pocket compared to conventional curettes and improve periodontal clinical parameters significantly [11, 22]. Our results revealed that the use of angled laser tip induces rough and irregular surface formation compared to the use of curette. In accordance to our findings, in 2002, Folwaczny et al. [4] reported that when Er:YAG laser was used in different parameters and tip angles, the increase of surface roughness was similar to that of non-treated root surfaces. Additionally, no significant differences were detected between different tip angulations and parameters. It is known from the literature that surface roughness increases plaque accumulation at the coronal region of the root surface, whereas it is stated to enhance fibroblast attachment at the middle and apical portions and may positively affect wound healing [10, 21, 22]. 

The usage of bovine teeth in dental research has been discussed in the literature which is the most commonly chosen substitute of human teeth, since they are easily obtained and the ethics committees are encouraging their use as an alternative for human teeth [23]. Previous studies have reported that due to the similarities of bovine and human dentine in terms of histomorphological surface characteristics and hardness, bovine teeth may be used as an alternative to the extracted human teeth [24, 25]. Therefore, freshly extracted, non carious bovine teeth which is kept in 0.2% thymol solution was used in the present study. In order to evaluate root surface morphology and roughness, the use of profilometer is a frequently-preferred method in studies since it has the advantages of being practical, requiring no previous preparation and giving quick results [5, 10, 15, 20, 26]. However, profilometric results alone are not reliable in evaluating treatment efficacy as they cannot measure the roughness of the area outside the contact tip [15]. The results must be evaluated together with SEM images as well. In the present study, a profilometer was used to evaluate the surface roughness of the samples, and the findings were supported by examining representative samples from each treatment group by SEM in order to evaluate the surfaces outside the contact area of the profilometer tip. In the literature, many different methods other than SEM and profilometers have been used to analyze the surface alterations of tooth-dental materials following the application of different instruments such as a three-dimensional optic laser scanner, an atomic force microscope, histological evaluation, and a digital stereomicroscope [13, 18, 19]. There has been no gold-standard method that can provide comprehensive surface assessment, and each approach has its own limitations [27]. 

During the instrumentation process, the lack of specific lateral force standardization may be regarded as a limitation of the study, which has also been reported by previous studies as its implementation is not easily replicable and standardized [18, 19]. 

In conclusion, the findings of the study revealed that the appropriate use of new generation ultrasonic tips creates a biologically acceptable root surface. Current findings need to be supported by further different cell culture studies evaluating bacterial and cellular adhesion as well as clinical studies examining periodontal parameters.

## Clinical relevance

Root surface debridement has a pivotal role in non-surgical and surgical periodontal therapies as well as supportive periodontal therapy phase. Although many instruments are available to disrupt and remove the acquired biofilm, there is no clear-cut consensus regarding their treatment efficacy. Therefore, preliminary data obtained from the present study may serve as the basis for upcoming clinical trials evaluating the patient-related outcomes of the usage of new-generation ultrasonic tips together with the periodontal clinical parameters.
